# Noncanonical, Dopamine-Dependent Long-Term Potentiation at Hippocampal Output Synapses in a Rodent Model of First-Episode Psychosis

**DOI:** 10.3389/fnmol.2020.00055

**Published:** 2020-04-03

**Authors:** Julia C. Bartsch, Joachim Behr

**Affiliations:** ^1^Department of Psychiatry and Psychotherapy, Charité-Universitätsmedizin Berlin, Berlin, Germany; ^2^Department of Psychiatry, Psychotherapy and Psychosomatic Medicine, Brandenburg Medical School, Neuruppin, Germany

**Keywords:** hippocampus, schizophrenia, synaptic plasticity, subiculum, psychosis, long-term potentiation, MK-801, dopamine

## Abstract

Cognitive deficits and positive symptoms in schizophrenia have both been linked to hippocampal dysfunction. Recently, subregion-specific aberrant and maladaptive hippocampal synaptic plasticity has been suggested as one of the mechanistic underpinnings. The subiculum is the final output hub of the hippocampus and orchestrates hippocampal information transfer to other brain regions. While most CA1 pyramidal neurons show regular-spiking behavior, subicular output neurons comprise bursting and regular-firing pyramidal cells. These two cell types target different brain regions and express unique forms of synaptic plasticity. Here, we used a single systemic application of the noncompetitive glutamatergic N-methyl-D-aspartate receptor (NMDAR) antagonist MK-801 to model first-episode psychosis in rats and studied long-term potentiation (LTP) in subicular regular-firing cells in acute hippocampal slices. Previously, we have reported a facilitation of a presynaptic, late-onset LTP in subicular bursting pyramidal cells after systemic NMDAR antagonism. Here, we show that single systemic NMDAR antagonist application also facilitates the induction of a noncanonical, but postsynaptic NMDAR-independent LTP in ventral subicular but not in CA1 regular-firing pyramidal cells. This form of LTP was dependent on D1/D5 dopamine receptor activation. Activation of D1/D5 dopamine receptors by a specific agonist mimicked and occluded LTP induced by electrical high-frequency stimulation (HFS). Furthermore, our results indicate that this form of LTP relies on postsynaptic Ca^2+^ signaling and requires the activation of protein kinase A. Considering the pivotal role of the subiculum as information gatekeeper between the hippocampus and other brain regions, this aberrant LTP in ventral subicular regular-firing neurons is expected to interfere with physiological hippocampal output processing and might thereby contribute to hippocampal dysfunction in psychotic events.

## Introduction

Roughly one percent of the global population is affected by schizophrenia. This multifaceted, heterogeneous psychiatric disorder is characterized by positive symptoms, i.e., delusions or hallucinations, negative symptoms, e.g., anhedonia, and cognitive symptoms including aberrant hippocampus-dependent memory encoding and retrieval as well as novelty detection (Heckers et al., 1998; Zierhut et al., 2010; Tamminga et al., 2012b; Pirnia et al., 2015; Ragland et al., 2015; Schott et al., 2015). In light of the accumulating evidence from large-scale genetic studies (Hall et al., 2015), contemporary pathophysiological concepts of schizophrenia suggest aberrations of synaptic plasticity are core to schizophrenia (McGlashan, 2006; Averbeck and Chafee, 2016). Synaptic plasticity in neuronal networks is regarded as one of the underpinning mechanisms for learning and memory enabling the individual to cope with a changing environment. Long-term potentiation (LTP) is one form of synaptic plasticity, defined by a lasting increase in synaptic efficacy following prior activity (Bliss and Lomo, 1973). Hippocampal dysfunction has been linked to both cognitive deficits and positive symptoms in schizophrenia (Bogerts, 1997) and hippocampal dysfunction in rodent model psychosis comprises subregion-specific maladaptive synaptic plasticity (Tamminga et al., 2012a). While no single animal model can completely match all aspects of an inherently human psychiatric disorder like schizophrenia, animal models are key to investigate the neurobiological mechanisms of schizophrenia at the cellular level (Averbeck and Chafee, 2016). Certain cognitive facets of schizophrenia can satisfactorily be modeled and studied in rodents by administering antagonists of the glutamatergic N-methyl-D-aspartate receptor (NMDAR, Neill et al., 2010). This rodent psychosis model originates from the NMDAR hypofunction concept which builds on the observation that acutely given NMDAR antagonists can induce psychosis in healthy humans similar to positive, negative and cognitive symptoms of schizophrenia (Luby et al., 1959). The hippocampal formation consists of the cornu ammonis (CA1–CA3 in rodents, hippocampus proper), the dentate gyrus and the subiculum. The dorsal hippocampus is mainly associated with spatial learning, while the ventral hippocampus is involved in emotion- and motivation-related processes (Fanselow, 2000; Bannerman et al., 2004; reviewed in Jarrard, 1995; Moser and Moser, 1998; Maren, 1999; Sharp, 1999; Fanselow and Dong, 2010; Segal et al., 2010; reviewed in Strange et al., 2014). The subiculum is the final output hub of the hippocampus and orchestrates hippocampal information transfer to other brain regions (O’Mara et al., 2000; Aggleton and Christiansen, 2015). Subicular pyramidal neurons can be distinguished in regular-spiking and burst-spiking neurons (Stewart and Wong, 1993; Taube, 1993; Behr et al., 1996; Mattia et al., 1997; Staff et al., 2000; Harris and Stewart, 2001; Wellmer et al., 2002), both show cell type-specific LTP (Wozny et al., 2008a,b) and target different brain regions. Previously, we have reported a facilitation of a presynaptic, late-onset LTP in subicular bursting pyramidal cells after systemic NMDAR antagonism (Bartsch et al., 2015). Here, we used a single systemic application of the noncompetitive NMDAR antagonist MK-801 to model psychosis in rats and studied LTP specifically in ventral subicular regular-firing cells in acute hippocampal slices.

## Materials and Methods

### Animals

All experiments were carried out under the directive 2010/63/EU of the European Parliament and of the council of 22 September 2010. Ethical approval for all rats used in this study was obtained from the Landesamt für Gesundheit und Soziales Berlin (LAGeSo Berlin, Germany). Rats were kept on a 12 h light-dark cycle and had access to food and water *ad libitum*. Wistar rats of both sexes (4–6 weeks old) received a single intraperitoneal injection with either MK-801 [(5S,10R)-(+)-5-methyl-10,11-dihydro-5H-dibenzo(a,d)cyclohepten-5,10-imine maleate, 5 mg/kg body weight], or 0.9% saline (10 ml/kg body weight).

### Slice Preparation

Twenty-four hours after i.p. injection, rats were decapitated under deep anesthesia (isoflurane, 1-chloro-2,2,2-trifluoroethyl-difluoromethylether; 2.5% in O_2_) and the brains were quickly removed. Horizontal slices (350–400 μm) containing the hippocampal formation and the entorhinal cortex were obtained with a Leica VT1200S vibratome (Leica Microsystems CMS, Mannheim, Germany). Slices were collected from the ventral sector of the hippocampus (~2 mm of the temporal pole), avoiding the extreme 350 μm end (Maggio and Segal, 2007a). Tissue for sharp microelectrode recordings was prepared in ice-cold, oxygenated (95% O_2_, 5% CO_2_) artificial cerebrospinal fluid (ACSF) and the slices were transferred for storage to an interface recording chamber continuously perfused (1.5–2 ml/min) with oxygenated and prewarmed (34°C) ACSF. The composition of the ACSF was as follows (in mM): NaCl 129, Na_2_PO_4_ 1.25, NaHCO_3_ 26, KCl 3, CaCl_2_ 1.6, MgSO_4_ 1.8, glucose 10 at a pH of 7.4. Slices for patch-clamp recordings were prepared in ice-cold, saccharose-based ACSF (in mM): NaCl 87, Na_2_PO_4_ 1.25, NaHCO_3_ 26, KCl 2.5, CaCl_2_ 0.5, MgCl_2_ 7, saccharose 75, glucose 25 at a pH of 7.4. After preparation, slices were kept under submerged conditions at 35°C for approximately 30 min and were then transferred to physiological ACSF solution at room temperature for further storage.

### Electrophysiology

Single-cell recordings in ventral CA1 pyramidal cells and regular-firing pyramidal cells of the subiculum were performed with sharp microelectrodes (40–100 MΩ) filled with 2.5 M potassium acetate in current-clamp bridge mode at resting membrane potential. Patch-clamp recordings were performed under submerged conditions at room temperature in voltage-clamp mode at a holding potential of −70 mV. Patch-clamp electrodes (4–6 MΩ) were filled with (in mM): K-gluconate 135, KCl 20, HEPES 10, phosphocreatine 7, Mg-ATP 2, Na-GTP 0.3, EGTA 0.2 and adjusted with KOH to a pH of 7.2. Access resistance was monitored throughout the experiments and recordings with fluctuating access resistance were discarded. No series resistance compensation was used. Signals were low-pass filtered at 3 kHz, sampled at 10 kHz by an ITC-16 interface (Instrutech Corp., Great Neck, NY, USA) and processed by TIDA software (HEKA GmbH, Lambrecht, Germany).

To study synaptic plasticity at glutamatergic synapses, all experiments were performed in the presence of bicuculline (5 μM) to block GABA_A_ receptor-mediated responses. To prevent polysynaptic responses, concentrations of MgSO_4_ and CaCl_2_ were elevated to 4 mM each (Nicholls and Purves, 1970; Berry and Pentreath, 1976; Wigström and Gustafsson, 1983; Miles and Wong, 1987; Wozny et al., 2008a). Excitatory postsynaptic potentials or currents (EPSP/EPSC) were evoked at 0.1 Hz by stimulation (100 μs) of Schaffer collaterals or CA1 efferents with an ACSF-filled patch pipette or a bipolar stimulating electrode. Baseline responses were recorded for at least 10 min and the stimulus intensity was set to evoke amplitudes of 30–50% of the maximum response. Depending on the rationale of the experiment, different high-frequency stimulation (HFS) protocols were used for the induction of LTP. Two-hundred pulses at 50 Hz, 25 pulses at 50 Hz, or 10 pulses at 40 Hz. Changes in synaptic strength were measured for 30 min after induction. Amplitudes of evoked EPSP or EPSC were normalized to baseline values. LTP was calculated by averaging the responses collected during the last 5 min of each experiment. Paired-pulse index (PPI) was investigated by analyzing the ratio of the second to the first synaptic response (EPSP2/EPSP1) at an inter-stimulus interval of 60 ms (Zucker and Regehr, 2002).

### Data Analysis/Statistics

Electrophysiological data were analyzed offline with Clampfit software (Molecular Devices Corporation, Sunnyvale, CA, USA), TIDA software (HEKA GmbH, Lambrecht, Germany) and GraphPad Prism (GraphPad Software, La Jolla, CA, USA). All datasets were tested for significant outliers using the Grubbs’ test (significance level, *p* < 0.05). Data are presented as mean ± standard error of the mean (SEM) or box and whisker plots (box: 25th to 75th percentiles, whiskers 5th and 95th percentiles). In figures illustrating the experimental time course of normalized amplitudes, data points were binned from six consecutive responses. Representative traces are shown as averages of six responses with clipped stimulation artifacts. The significance level was set to *p* < 0.05. Comparisons within groups were analyzed by paired Student’s *t*-tests. Multigroup comparisons were analyzed with the Kruskal–Wallis test followed by Dunn’s *post hoc* test against the control group (Figures 2F, 3E). Numbers given in the text (x/y) refer to numbers of neurons (x) recorded in different animals (y).

**Figure 1:**
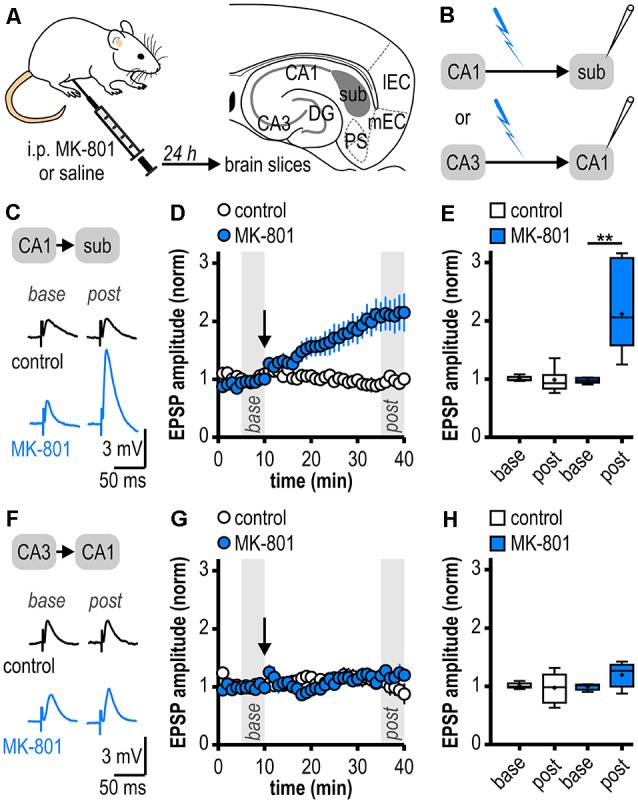
Facilitated long-term potentiation (LTP) in ventral subicular regular-firing neurons but not in CA1 pyramidal neurons following systemic N-methyl-D-aspartate receptor (NMDAR) antagonism. **(A)** Study design. Schematic drawing of a horizontal section through the ventral rat hippocampal formation with distinct subregions: medial and lateral entorhinal cortex (mEC, lEC); dentate gyrus (DG); cornu ammonis 1 and 3 (CA3, CA1); subiculum (sub); pre- and parasubiculum (PS). **(B)** Scheme of the experimental approach to study LTP induction at ventral hippocampal output synapses using intracellular recordings with sharp microelectrodes. **(C)** Representative traces of excitatory postsynaptic potentials (EPSPs) recorded in subicular regular-firing neurons before (*base*, min 6–10) and after (*post*, min 36–40) application of brief high-frequency stimulation (HFS; 10 pulses at 40 Hz). **(D)** Time course of mean normalized EPSP amplitudes at CA1–subiculum synapses to regular-firing pyramidal neurons (control: *n* = 7/6, MK-801: *n* = 7/5). HFS (arrow) induces robust LTP only in MK-801-treated rats. **(E)** Quantification of mean normalized EPSP amplitudes (control: *n* = 7/6, paired Student’s *t*-test *p* = 0.78; MK-801: *n* = 7/5, paired Student’s *t*-test **p < 0.01). **(F)** Representative traces of EPSPs recorded in CA1 pyramidal neurons before (*base*, min 6–10) and after (*post*, min 36–40) application of brief HFS (25 pulses at 50 Hz). **(G)** Time course of mean normalized EPSP amplitudes at Schaffer collateral input to CA1 pyramidal cells. HFS (arrow) fails to induce LTP in both animal groups. **(H)** Quantification of mean normalized EPSP amplitudes (control: *n* = 6/5, paired Student’s *t*-test *p* = 0.78; MK-801: *n* = 5/4, paired Student’s *t*-test *p* = 0.15).

**Figure 2:**
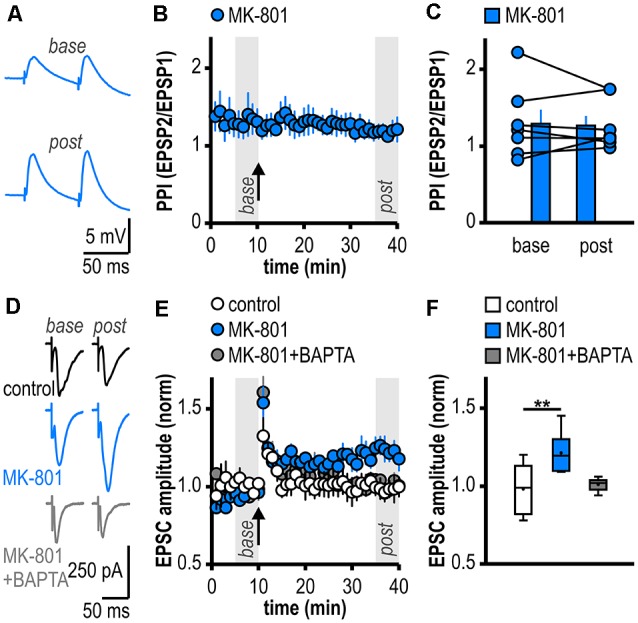
Facilitated LTP in regular-firing subicular neurons of MK-801-treated rats is expressed postsynaptically and is dependent on postsynaptic Ca^2+^. **(A)** Representative traces of paired EPSPs (60 ms inter-stimulus interval) recorded in subicular regular-firing neurons of MK-801-treated rats before (*base*, min 6–10) and after (*post*, min 36–40) application of HFS. **(B)** Experimental time course of paired-pulse index (PPI). PPI does not change after the induction of LTP in MK-801-treated rats. **(C)** Quantification of PPI. Filled circles depict the mean value of single neurons before (*base*) and after (*post*) LTP induction, columns represent mean ± SEM of all recorded neurons (*n* = 7/5, paired Student’s *t*-test *p* = 0.79). **(D)** Representative traces of excitatory postsynaptic currents (EPSCs) recorded in subicular regular-firing neurons before (*base*, min 6–10) and after (*post*, min 36–40) application of brief HFS (200 pulses at 50 Hz) using the whole-cell patch-clamp technique. **(E)** Postsynaptic dialysis with the Ca^2+^ buffer BAPTA prevents the expression of LTP in MK-801-treated rats (control: *n* = 7/5, MK-801: *n* = 6/6, MK-801+BAPTA: *n* = 6/4). **(F)** Quantification of mean normalized EPSC amplitudes (Kruskal–Wallis test: *H*_(2)_ = 11.01, *p* = 0.0041, **p < 0.01 by *post hoc* Dunn).

**Figure 3:**
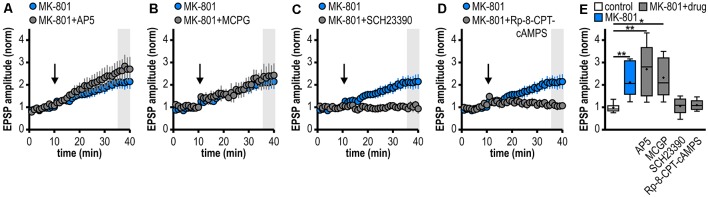
Noncanonical LTP in MK-801-treated rats depends on the activation of D1/D5R and the adenylate cyclase-cAMP-PKA signaling cascade but is independent from NMDAR and mGluRI/II. **(A–D)** Time course of mean normalized EPSP amplitudes at CA1–subiculum synapses to regular-firing pyramidal neurons under the bath-application of denoted drugs. MK-801 data taken from Figure 1D is replotted for comparison. **(A)** The NMDAR antagonist AP5 failed to prevent LTP in MK-801-treated rats (*n* = 6/5, paired Student’s *t*-test *p* < 0.05). **(B)** The mGluRI/II antagonist MCGP could not block LTP induction (*n* = 5/5, paired Student’s *t*-test *p* < 0.05). **(C)** The specific D1/D5R antagonist SCH23390 blocked LTP-induction (*n* = 6/4, paired Student’s *t*-test *p* = 0.64). **(D)** Preincubation of slices with the PKA inhibitor Rp-8-CPT-cAMPS prevented the induction of LTP (*n* = 5/3, paired Student’s *t*-test *p* = 0.68). **(E)** Summary of changes in synaptic strength following brief HFS for the different experimental groups shown in **(A–D)**. Control and MK-801 data taken from Figure 1E is replotted for comparison. Quantification of mean normalized EPSP amplitudes (Kruskal–Wallis test: *H*_(5)_ = 23.18, *p* = 0.0003, *p < 0.05, **p < 0.01 by *post hoc* Dunn).

### Drugs

The following drugs were used: (+)-MK-801 maleate [(5S,10R)-(+)-5-methyl-10,11-dihydro-5H-dibenzo(a,d)cyclohepten-5,10-imine maleate], (-)-bicuculline methiodide ((R*-*(R*,S*))-5-(6, 8-dihydro-8-oxofuro(3,4-e)-1,3-benzodioxol-6-yl)-5,6,7,8-tetrahydro-6,6-dimethyl-1,3-dioxolo(4,5-g)isoquinolinium iodide), 5 μM; SCH23390 hydrochloride ((R)-(+)-7-chloro-8-hydroxy-3-methyl-1-phenyl-2,3,4,5-tetrahydro-1H-3-benzazepine hydrochloride), 10 μM; SKF38393 hydrobromide ((±)-1-phenyl-2,3,4,5-tetrahydro-(1H)-3-benzazepine-7,8-diol hydrobromide), 100 μM; D-AP5 (D-(-)-2-amino-5-phosphonopentanoic acid), 100 μM; (RS)-MCPG ((RS)-α-methyl-4-carboxyphenylglycine), 500 μM; BAPTA (1,2-bis(2-aminophenoxy)ethane-N,N,N′,N′-tetraacetic acid), 30 mM in the intracellular solution; Rp-8-CPT-cAMPS (8-(4-chlorophenylthio)adenosine-3′,5′-cyclic monophosphorothioate), 100 μM. Drugs were purchased from Biolog, Germany; Sigma–Aldrich, Germany; Ascent Scientific, UK and Tocris, UK. Most drugs were bath-applied for at least 10 min before starting the experiment. In experiments using the PKA inhibitor Rp-8-CPT-cAMPS, slices were incubated for at least 1 h before commencement of recordings. Loading recorded neurons with the calcium chelator BAPTA did not alter the discharge behavior of subicular pyramidal cells. Stable baseline responses could be obtained in most of the recordings with BAPTA. Cells that showed a rundown of responses (~15%, Lapointe et al., 2004) were not included.

## Results

### Facilitated LTP in Ventral Subicular but Not in CA1 Pyramidal Neurons Following Systemic NMDAR Antagonism

As systemic NMDAR antagonism has been reported to facilitate a noncanonical late-onset LTP at CA1-subiculum synapses to bursting pyramidal cells (Bartsch et al., 2015), we first studied whether this holds also true for ventral CA1-subiculum synapses to regular-firing pyramidal cells. Recordings were performed in regular-firing pyramidal cells in the middle portion of the subiculum concerning the proximodistal axis using sharp microelectrodes to leave the intracellular environment physiological (Figures 1A,B). We applied a brief HFS protocol (10 pulses, 40 Hz), which was insufficient to induce LTP at CA1–subiculum synapses to regular-firing pyramidal cells under control conditions (0.99 ± 0.08, *n* = 7/6, *p* = 0.78; Figures 1C–E). In MK-801-treated rats, however, the subthreshold HFS induced stable LTP appearing as a lasting late-onset increase in EPSP amplitudes in subicular regular-firing pyramidal cells (2.12 ± 0.28, *n* = 7/5, *p* < 0.01; Figures 1C–E). Previously, we have shown that this brief HFS does not induce LTP at CA3–CA1 synapses in MK-801-treated rats (Bartsch et al., 2015). Here, we also tested an adapted, slightly stronger brief HFS protocol (25 pulses, 50 Hz) set subthreshold for LTP induction in control rats in CA1 pyramidal cells. In line with our previous results, this adapted brief HFS also failed to induce LTP (control: 0.98 ± 0.11, *n* = 6/5, *p* = 0.78; MK-801: 1.20 ± 0.10, *n* = 5/4, *p* = 0.15; Figures 1F–H). Hence, we conclude that this form of LTP is exclusive to subicular regular-firing pyramidal neurons.

### Facilitated LTP in Regular-Firing Subicular Neurons of MK-801-Treated Rats Is Expressed Postsynaptically and Is Dependent on Postsynaptic Ca^2+^

To investigate the expression site of this facilitated LTP in regular-firing subicular neurons of MK-801-treated rats, we analyzed the PPI before and after LTP induction (Zucker and Regehr, 2002). The facilitated LTP in regular-firing neurons of MK-801-treated rats was not associated with significant changes in the PPI (base: 1.30 ± 0.18, LTP: 1.28 ± 0.12, *n* = 7/5, *p* = 0.79; Figures 2A–C), arguing for a postsynaptic expression site. In the next step, we determined if postsynaptic Ca^2+^ signaling is required for this facilitated LTP in regular-firing neurons of MK-801-treated rats. To this end, we performed whole-cell patch-clamp recordings to guarantee good dialysis of cells with a Ca^2+^ buffer. To replicate the LTP in MK-801-treated animals under patch-clamp conditions, we adjusted the HFS to 200 pulses at 50 Hz applied in current-clamp mode. This stimulation protocol induced LTP in MK-801-treated rats (1.21 ± 0.05, *n* = 6/6, *p* < 0.01; Figures 2D–F), but led to only brief post-tetanic potentiation and no LTP in control animals (1.01 ± 0.02, *n* = 7/5, *p* = 0.68; Figures 2D–F). As previously reported (Wozny et al., 2008b; Bartsch et al., 2015), in whole-cell patch-clamp recordings under submerged conditions LTP was smaller than in sharp microelectrode recordings under interface condition. Of note, LTP in patch-clamp recordings under submerged conditions exhibited a different time course lacking the late-onset component. A reasonable explanation for this observation might be a difference in oxygen supply under both recording conditions (Hájos et al., 2009). Postsynaptic dialysis of individual regular-firing neurons with the fast Ca^2+^ buffer BAPTA prevented the induction of LTP in MK-801-treated rats (1.01 ± 0.02, *n* = 6/4, *p* = 0.56; Figures 2D–F), suggesting that this facilitated LTP in subicular regular-firing neurons after systemic NMDAR antagonism is dependent on postsynaptic Ca^2+^ signaling.

### Noncanonical LTP in MK-801-Treated Rats Depends on the Activation of D1/D5R and the Adenylate Cyclase-cAMP-PKA Signaling Cascade but Is Independent of NMDAR and mGluRI/II

At various synapses in the central nervous system, LTP depends on NMDAR activation (Nicoll and Malenka, 1999; Morris, 2013; Volianskis et al., 2015). Also, subicular regular-firing neurons express NMDAR-dependent LTP (Wozny et al., 2008b). However, the application of the NMDAR antagonist AP5 failed to prevent the aberrant LTP in MK-801-treated rats (2.70 ± 0.49, *n* = 6/5; Figures 3A,E). This suggests that this late-onset LTP after systemic MK-801 treatment is NMDAR-independent. To test the involvement of metabotropic glutamate receptors (mGluR) in this NMDAR-independent LTP (O’Leary and O’Connor, 1999), we bath-applied the mGluRI/II antagonist MCGP. Again, this could not prevent LTP induction in regular-firing neurons from MK-801-treated rats (2.32 ± 0.40, *n* = 5/5; Figures 3B,E). This argues against a major involvement of mGluR in this aberrant form of LTP.

Previously, we have reported a facilitation of a presynaptic, late-onset LTP in subicular bursting pyramidal cells after systemic NMDAR antagonism that is induced by D1/D5 dopamine receptor (D1/D5R) activation (Bartsch et al., 2015). Thus, we tested if the facilitated LTP in subicular regular-firing neurons is also mediated by D1/D5R. Indeed, in the presence of the specific D1/D5R antagonist SCH23390, LTP-induction was blocked (1.06 ± 0.15, *n* = 6/4; Figures 3C,E). D1/D5R are G protein-coupled receptors that stimulate the adenylate cyclase-cAMP-protein kinase A (PKA) signaling cascade (Missale et al., 1998). Consequently, we investigated whether blocking this pathway prevents the induction of LTP in MK-801-treated animals. To this end, hippocampal brain slices were incubated with the PKA inhibitor Rp-8-CPT-cAMPS for at least 1 h before recording. Indeed, this treatment blocked the induction of LTP in MK-801-treated animals (1.10 ± 0.11, *n* = 5/3; Figures 3D,E), indicating that this form of LTP needs the activation of PKA. A summary of changes in synaptic strength in the reported experiments is shown in Figure 3E.

### D1/D5R Activation Mimics and Occludes Noncanonical HFS-Induced LTP in MK-801-Treated Rats

**Figure 4:**
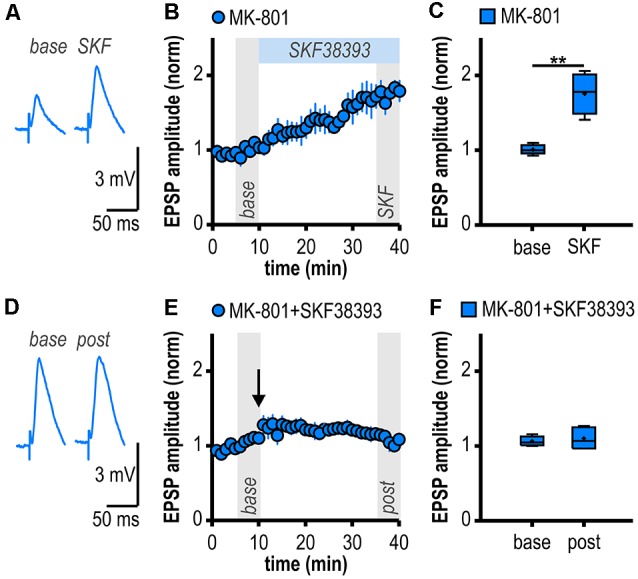
D1/D5R activation mimics and occludes noncanonical HFS-induced LTP in MK-801-treated rats. **(A)** Representative traces of EPSPs recorded in subicular regular-firing neurons before (*base*, min 6–10) and after (*SKF*, min 36–40) application of the D1/D5R agonist SKF38393. **(B)** Time course of mean normalized EPSP amplitudes at CA1–subiculum synapses to regular-firing pyramidal neurons. D1/D5R activation enhanced EPSPs in subicular regular-firing pyramidal neurons in MK-801-treated rats. **(C)** Quantification of mean normalized EPSP amplitudes (*n* = 5/4, paired Student’s *t*-test, **p < 0.01). **(D)** Representative traces of EPSPs recorded in subicular regular-firing neurons before (*base*, min 6–10) and after (*post*, min 36–40) application of brief HFS following D1/D5R activation. **(E)** SKF38393-induced enhancement of EPSPs prevented HFS-induced LTP (continued recordings of the same cells shown in **B**). **(F)** Quantification of mean normalized EPSP amplitudes (paired Student’s *t*-test, *p* = 0.52).

If D1/D5R activation is crucial for the aberrant LTP in MK-801-treated rats, activating these receptors by a specific agonist should also result in LTP. Accordingly, bath application of the specific D1/D5R agonist SKF38393 resulted in an enhancement of synaptic transmission in subicular regular-firing neurons of MK-801 treated rats (1.76 ± 0.12, *n* = 5/4, *p* < 0.01; Figures 4A–C) which mimicked and finally occluded tetanus-induced LTP (1.10 ± 0.06, *n* = 5/4, *p* = 0.52; Figures 4D–F). This shows that chemically and electrically induced LTP share common mechanisms.

## Discussion

The present findings confirm and extend previous studies on aberrant hippocampal LTP in a rodent model of acute psychosis. We show that single systemic NMDAR antagonism in young adult Wistar rats facilitates LTP induction at ventral CA1-subiculum synapses to regular-firing pyramidal cells tested *ex vivo* 1 day later. This noncanonical, late-onset LTP in MK-801-treated rats is postsynaptic, NMDAR-independent and relies on the activation of D1/D5R, PKA, and postsynaptic Ca^2+^ signaling.

### Subregion-Specific Changes in Hippocampal LTP in the MK-801 Rodent Model of First-Episode Psychosis

Previous e*x vivo* studies using acute brain slices have shown that a single systemic application of MK-801 can impair LTP at the Schaffer collateral input to CA1 (Wöhrl et al., 2007; Bartsch et al., 2015). Likewise, LTP at the direct cortical input to CA1 was reduced for prolonged periods (Wöhrl et al., 2007). Accordingly, we here also found no facilitation of LTP at the Schaffer collateral input to CA1. The facilitated LTP was exclusive to regular-firing subicular neurons. In line with our results, *ex vivo* field potential recordings at CA1-subiculum synapses indicated facilitated LTP 24 h after a single systemic application of MK-801 (Buck et al., 2006). Therefore, our and previous results argue for subregion-specific patterns of psychosis-induced changes in hippocampal LTP. Notably, the facilitation of LTP seems to be restricted to subicular hippocampal output synapses. The distinct propensity of subicular pyramidal cells to successfully express LTP might depend on various presynaptic and postsynaptic factors as the total number of synaptic contacts, presynaptic and postsynaptic Ca^2+^ influx, the number of release-competent vesicles or the desensitization state of postsynaptic receptors (Schneggenburger et al., 2002) and has to be delineated in more detail in future studies.

### Cell Type-Specific Aberrant LTP at Hippocampal Output Synapses in the MK-801 Rodent Model of First-Episode Psychosis

Adding up to the complexity, while most CA1 pyramidal neurons show regular-spiking behavior, subicular output neurons comprise bursting and regular-firing pyramidal cells (Stewart and Wong, 1993; Taube, 1993; Behr et al., 1996; Mattia et al., 1997; Staff et al., 2000; Harris and Stewart, 2001; Wellmer et al., 2002). These two cell types target different brain regions. Bursting pyramidal cells project to the presubiculum, the medial entorhinal cortex, the hypothalamus, and the retrosplenial cortex while regular-firing pyramidal cells target the lateral entorhinal cortex, the nucleus accumbens and the amygdala (Köhler, 1985; Stewart, 1997; Kim and Spruston, 2012). Interestingly, CA1 efferents to subicular pyramidal cells innately express cell type-specific forms of synaptic plasticity (Fidzinski et al., 2008; Wozny et al., 2008a,b; Aoto et al., 2013). In the MK-801 rodent model of first-episode psychosis, we have previously reported a facilitated late-onset LTP restricted to glutamatergic synapses between CA1 and subicular bursting pyramidal cells (Bartsch et al., 2015). This form of subicular LTP is expressed presynaptically. Here, we show that single systemic MK-801 application also facilitates the induction of a noncanonical, but postsynaptic LTP at glutamatergic CA1-subiculum synapses to regular-firing neurons in the ventral hippocampus: buffering postsynaptic Ca^2+^ signals prevented LTP induction in regular-firing subicular neurons and, in contrast to LTP at CA1 inputs to bursting subicular neurons, LTP in regular-firing neurons was not accompanied by changes in the PPI. Apparently, cell type-specific forms of synaptic plasticity at CA1 inputs to subicular neurons appear in the MK-801 rodent model of first-episode psychosis as well. Two subsets of CA1 terminals with different susceptibility to express a presynaptic form of LTP seem to exist and determine the target-specificity of CA1 inputs to subicular neurons (Fidzinski et al., 2008; Wozny et al., 2008a; Wójtowicz et al., 2010). Cell type-specific forms of subicular LTP might be of important functional relevance. Theoretical and evidence-based theories imply that postsynaptic forms of LTP lead to an unconditional gain of synaptic transmission (Selig et al., 1999) whereas presynaptic forms of LTP redistribute existing synaptic efficacy and thereby alter the dynamics of synaptic transmission (Markram and Tsodyks, 1996). The specific role of both types of subicular neurons during behavior has yet to be determined.

### Dopamine-Dependent Long-Term Potentiation at Hippocampal Output Synapses in a Rodent Model of First-Episode Psychosis

Several lines of evidence support a D1/D5R-dependent induction mechanism of the facilitated LTP in subicular regular-firing neurons in the MK-801 rodent model of first-episode psychosis. First, blocking D1/D5R with a specific antagonist prevented LTP induction, second, activating D1/D5R with a specific agonist mimicked LTP in MK-801-treated rats and, finally, occluded HFS-induced LTP. This mechanism is consistent with the D1/D5R-dependent facilitated LTP in bursting subicular neurons in MK-801-treated rats (Bartsch et al., 2015) yet differs from it in terms of expression site. In MK-801-treated rats, LTP in regular-firing neurons is dependent on postsynaptic Ca^2+^ signaling while LTP in bursting neurons is not. Previous studies have already shown that D1/D5R activation facilitates either pre- or postsynaptic LTP depending on the subicular cell type (Roggenhofer et al., 2010, 2013). However, facilitated subicular LTP in the MK-801 rodent model of first-episode psychosis is noncanonical in that it is NMDAR-independent and exhibits a differential time course (late-onset) compared with canonical subicular LTP (initial strong post-tetanic potentiation decaying to a stable plateau).

Several origins of hippocampus-projecting dopaminergic fibers and a differential innervation along the hippocampal longitudinal axis have been described. Especially dopaminergic fibers arising from the ventral tegmental area and the substantia nigra pars compacta have been suggested to target the ventral hippocampal sector (reviewed in Edelmann and Lessmann, 2018). Notably, systemic NMDAR application can boost dopamine release in many brain regions (Deutch et al., 1987; Whitton et al., 1992; Breier et al., 1998). Relating to our findings, electrical HFS of the subiculum or regional NMDA application in the subiculum can initiate dopamine release from ventral tegmental area terminals projecting to the hippocampus (Blaha et al., 1997; Taepavarapruk et al., 2000; Floresco et al., 2001, 2003) that most prominently innervate CA1 and the subiculum (Verney et al., 1985). Hence, an increased hippocampal output after systemic MK-801 application represented by the aberrant noncanonical LTP in ventral subicular regular-firing neurons in this study may initiate a positive feedback in the hippocampus-ventral tegmental area loop and in turn, trigger an overdrive of the dopaminergic system. This scenario would be well in agreement with models proposing a hyperactive ventral hippocampus driving mesolimbic dopaminergic hyperfunction and resulting in aberrant dopaminergic signaling (Lodge and Grace, 2007). The distinct susceptibility of ventral subicular pyramidal cells to endogenously released dopamine could result from a region-specific distribution of dopamine receptor subtypes. However, D1R and D5R are expressed in pyramidal cells of the hippocampus proper and prominently in the ventral subiculum in rats (Fremeau et al., 1991). Immunohistochemical staining suggests that D5R mostly reside on cell bodies than on dendrites (Ciliax et al., 2000; Khan et al., 2000). Thus, the number of activated D1/D5R may determine differences in dopamine-dependent LTP facilitation in CA1 and the subiculum.

Considering the postsynaptic expression of LTP reported in this study, the most plausible mechanistic flow, therefore, seems to be the following: after systemic NMDAR antagonism, brief HFS can activate D1/D5R presumably *via* endogenous dopamine release. Activation of D1/D5R, in turn, elevates postsynaptic cAMP concentrations and initiates the cAMP-PKA cascade by a Gs-adenylate cyclase pathway in subicular regular-firing neurons (Missale et al., 1998). This increases postsynaptic Ca^2+^ signaling *via* NMDAR-independent mechanisms (Cepeda et al., 1993, 1998; Surmeier et al., 1995; Hernández-López et al., 1997) which could modulate the membrane potential, favor EPSP summation and thereby lower the induction threshold for LTP resulting in a facilitated LTP.

### Possible Functional Impact of the Aberrant Noncanonical LTP in Ventral Subicular Regular-Firing Neurons

A functional segregation along the longitudinal axis of the hippocampus has been suggested (Moser and Moser, 1998). The dorsal (septal) sector of the hippocampus, corresponding to the human posterior hippocampus, is mostly involved in spatial memory function while the ventral (temporal) pole of the hippocampus, corresponding to the human anterior hippocampus, modulates emotional, motivational and affective processes (reviewed in Fanselow and Dong, 2010; reviewed in Strange et al., 2014). Schizophrenia is associated with anterior hippocampal pathology (Titone et al., 2004; Öngür et al., 2006; Small et al., 2011; Lieberman et al., 2018). The ability to express LTP also varies along the septotemporal axis of the hippocampus: ventral CA1 expresses lower LTP compared with dorsal CA1 but this can change under pathophysiological conditions (Maggio and Segal, 2007a,b, 2011). Bearing in mind the pivotal role of the subiculum as information gatekeeper between the hippocampus and other brain regions, this aberrant LTP in ventral subicular regular-firing neurons is expected to interfere with physiological hippocampal output processing. Thereby, our results add to the notion from previous rodent studies suggesting that subregion-specific reduced synaptic plasticity or aberrant hyperplasticity contributes to hippocampal dysfunction in first-episode psychosis (Tamminga et al., 2012a; Bartsch et al., 2019). Moreover, our observation of a cell type-specific facilitation of subicular LTP after systemic NMDAR antagonism can be considered as a form of metaplasticity. Metaplasticity is defined as a lasting modification in the neuronal state following activation which can hence regulate the duration, magnitude or direction of future synaptic plasticity (Abraham and Bear, 1996; Hulme et al., 2013). Related to first-episode psychosis, this metaplasticity might be maladaptive and could subsequently lead to hyperplasticity in key brain circuits associated with psychosis and schizophrenia (hippocampus-ventral tegmental area; hippocampus-medial prefrontal cortex) resulting in network dysregulation. Such network dysregulation is expected to contribute to the pathophysiology and perpetuation of acute psychotic symptoms and schizophrenia.

## Data Availability Statement

The datasets generated and analyzed during the current study are available from the corresponding author on reasonable request.

## Ethics Statement

The animal study was reviewed and approved by Landesamt für Gesundheit und Soziales Berlin (LAGeSo Berlin, Germany).

## Author Contributions

JCB and JB were responsible for conception of the study, design of the experiments and interpretation of the data and approved the final version and therefore agree to be accountable for all aspects of the work. JCB acquired and analyzed the data and wrote the manuscript with inputs from JB.

## Conflict of Interest

The authors declare that the research was conducted in the absence of any commercial or financial relationships that could be construed as a potential conflict of interest.
